# Soft Tissue Sarcomas of the Thoracic Wall: More Prone to Higher Mortality, and Local Recurrence—A Single Institution Long-Term Follow-up Study

**DOI:** 10.1155/2019/2350157

**Published:** 2019-03-04

**Authors:** Tine Rytter Soerensen, Mathias Raedkjaer, Peter Holmberg Jørgensen, Anette Hoejsgaard, Akmal Safwat, Thomas Baad-Hansen

**Affiliations:** ^1^Department of Orthopaedic Oncology, Aarhus University Hospital, Denmark; ^2^Department of Cardiovascular Surgery, Aarhus University Hospital, Denmark; ^3^Department of Oncology and Danish Center for Particle Therapy, Aarhus University Hospital, Denmark

## Abstract

**Objectives:**

This study aims to assess the impact of surgical margin and malignancy grade on overall survival (OS) and local recurrence free rate (LRFR) for soft tissue sarcomas (STS) of the thoracic wall.

**Methods:**

This retrospective cohort study identified 88 patients, diagnosed and treated surgically for a nonmetastatic STS located in the thoracic wall between 1995 and 2013, using the population based and validated Aarhus Sarcoma Registry and Danish Sarcoma Registry. The Kaplan-Meier method was used to estimate OS and LRFR. Multivariate Cox analyses were used to determine prognostic factors for OS and LRFR.

**Results:**

The 5-year OS was 55% (95% confidence interval (CI): 0.44-0.65) and 5-year LRFR was 77% (95% CI: 0.67-0.85). High malignancy grade and intralesional/marginal resection were identified as negative predictors for OS. High grade was the only prognostic factor associated with a lower LRFR.

**Conclusions:**

In this large, single institution, study tumor grade was the key predictor for OS and LRFR. Surgical margin only statistically significantly influenced mortality, not local recurrence.

## 1. Introduction

Soft tissue sarcomas (STS) of the thoracic wall pose a clinical challenge due to their rarity and localization. Most previous studies describing chest wall STS have not distinguished between bone/cartilaginous tumors and STS or in combination with STS of the extremities [1–10]. Research on STS of the thoracic wall is limited to a few reports with small patient cohorts [11–16]. Literature data is consistent in showing that malignancy grade is an important prognostic factor regarding survival and local control for sarcomas [1–9, 17]. However, the impact of surgical margin is still debatable [2, 8, 11, 12, 14, 17–21].

The clinical behavior and the prognostic factors for sarcomas of the chest wall are usually assumed to be similar to extremity STS [1, 14, 16]. However, studies have indicated that the prognosis of sarcomas may depend on anatomical localization and a comparison of STS of the extremities with STS of the thoracic wall has shown a lower median survival of the latter [9, 18, 22, 23].

The aim of this study was to analyze our institutional experience with STS arising in the thoracic wall in terms of the impact of malignancy grade and surgical margin on mortality and local control.

## 2. Material and Methods

**Figure 1:**
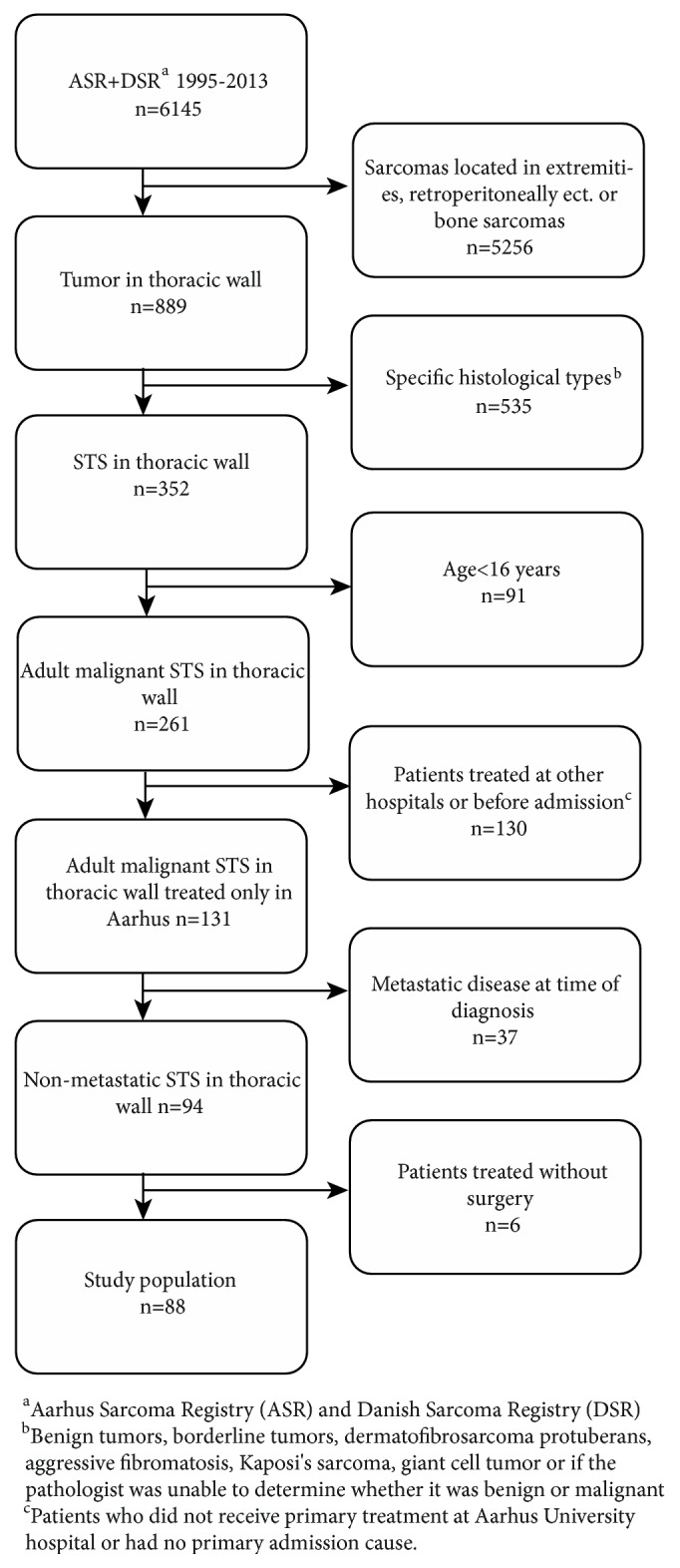
Flowchart of patients registered in Aarhus Sarcoma Registry (ASR) and Danish Sarcoma Registry (DSR) in the period 1995-2013. Number of patients (n), exclusion criteria, and the study population of adult patients treated surgically for a soft tissue sarcoma (STS) in the thoracic wall.

The study cohort consisted of patients undergoing surgical treatment for a localized nonmetastatic STS of the thoracic wall in the period 1995-2013 at Aarhus University Hospital (ASC). Patients below 16 years of age and certain histological types were excluded (Figure 1).

The cohort was identified using the Aarhus Sarcoma Registry (ASR) and after 2009 the Danish Sarcoma Registry (DSR). ASR and DSR are population based and validated and contain information on patient demographics, tumor-specific data regarding size, localization, and malignancy grade as well as information about treatment and follow-up examinations including local recurrence (LR), distant metastasis and death.

Treatment is standardized according to international ESMO guidelines for STS [24]. Surgery is the main treatment, aiming at wide margins [24]. The surgical margin was defined based on the classification of Enneking [25]. Intralesional and marginal resection were joined into a single group, since preliminary analysis did not reveal any difference between the two groups in terms of mortality and local control. Myhre Jensen and the French Federation of Comprehensive Cancer Centers (FNCLCC) scales were used to classify the resected tumor into low-, intermediate-, or high-grade based on cellularity, mitotic activity, anaplasia, and necrosis [26, 27]. Intermediate- and high-grade tumors were combined into one group, defined as “high grade” in this study, as the oncological treatment is similar among the two groups: postoperative radiotherapy for intermediate- and high-grade tumors and deep-seated tumors, with the exception of individuals affected with small tumors, elderly, or frail patients [2]. Postoperative radiotherapy is given as a daily dose of 2 Gray (Gy) to a total dose of 50-66 Gy. Chemotherapy is not standard treatment but is offered based on an individual assessment.

### 2.1. Statistical Analysis

Overall survival (OS) calculated from time of diagnosis and local recurrence free rate (LRFR) were estimated using the Kaplan-Meier method. Possible prognostic factors were included in a Cox proportional hazards model for univariate and multivariate analysis, to assess their association with OS and LRFR. A P-value below 0.05 was considered significant. STATA software 14.1 was used to perform the statistical analysis.

### 2.2. Ethics

The study was approved by the Danish Data Protection Agency (j.nr: 1-16-02-245-14) and the Danish Clinical Registries (j.nr: DSD-2017-03-02).

## 3. Results

### 3.1. Patient and Tumor Characteristics

Between January 1st, 1995, and December 31st, 2013, a total of 88 patients were treated surgically for a localized nonmetastatic STS of the thoracic wall (Figure 1).

Mean age at diagnosis was 56 (range 16-86) years and 58% were males (Table 1). Undifferentiated pleomorphic sarcoma (UPS) previously malignant fibrous histiocytoma (MFH) and leiomyosarcoma were the most frequent types (Table 2).

A total of 11 patients (13%) presented with a low grade tumor, seven of them (64%) were treated with wide resection, and four (36%) with intralesional/marginal resection. None of the patients with low grade tumors received radiotherapy or chemotherapy. None of the patients with low grade tumors developed LR or distant metastases.

The majority, 77 patients (87%), had a high grade tumor; within this group, wide resection was achieved in 51 patients (66%). There were 30 patients with high grade tumors who received postoperative radiotherapy, 18 of them (60%) had wide resection and 12 (40%) had intralesional/marginal resection. A total of 13 patients were treated with chemotherapy, two (15%) were treated with wide resection, and 11 (85%) had an intralesional/marginal resection. LR occurred in 18 cases (20%) and five of these patients also developed distant metastasis. A total of 18 patients (20%) with high grade tumors developed distant metastases.

In the observation period 44 patients (50%) died. Tumor-related death occurred in 29 cases; 15 patients died of other causes, not related to the sarcoma.

Mean follow-up was 79 months (range 2-249 months).

**Figure 2:**
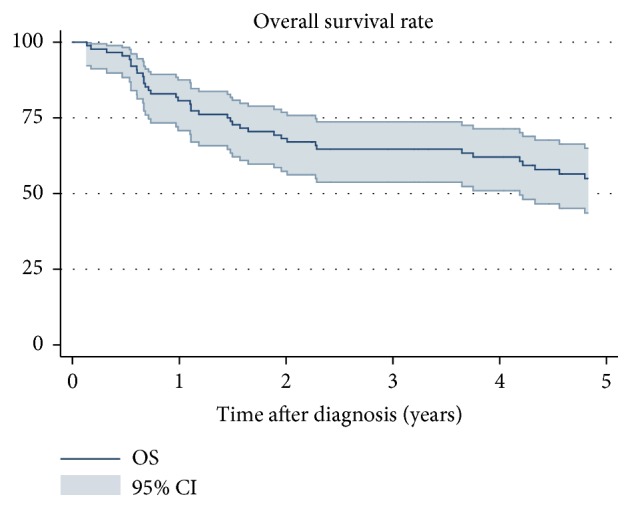
Survival rate of 88 patients with soft tissue sarcoma in the thoracic wall. 95% confidence intervals.

**Figure 3:**
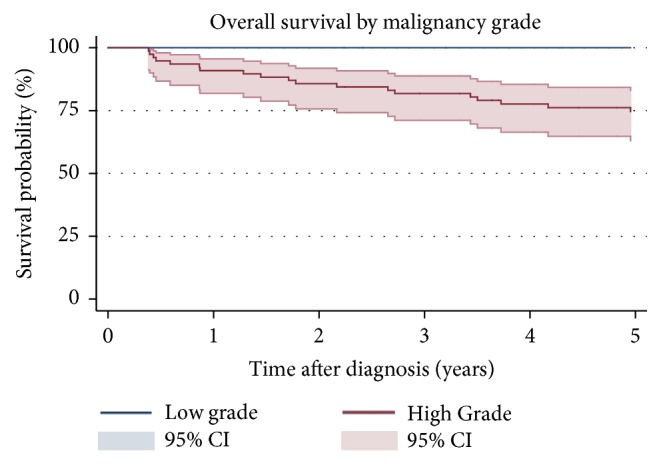
Kaplan-Meier estimates of overall survival by malignancy grade. 95% confidence intervals.

**Figure 4:**
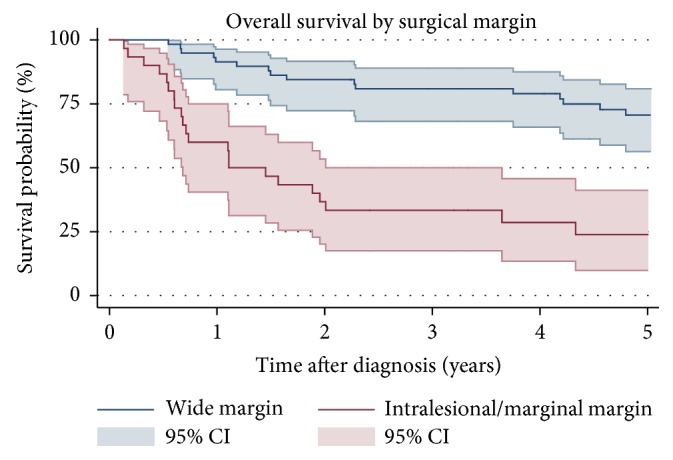
Kaplan-Meier estimates of overall survival by surgical margin. 95% confidence intervals.

The 5-year OS, 55% (95% confidence interval (CI): 0.44-0.65), is shown in Figure 2. Patients with high grade tumors (5-year OS 49%, 95% CI: 0.37-0.60) had a worse 5-year OS compared to patients with low grade tumors (Figure 3). Wide margins (5-year OS: 0.71 95% CI: 0.56-81) correlated with a better 5-year OS than intralesional/marginal resection (5-year OS 24%, 95% CI: 0.10-0.41) (Figure 4). The results of the univariable and multivariable analysis are shown in Table 3. High grade was a negative prognostic factor for OS as none of the patients with low grade tumors died. Intralesional/marginal resection (HR 3.06, 95% CI: 1.24-7.53) was associated with worse OS.

**Figure 5:**
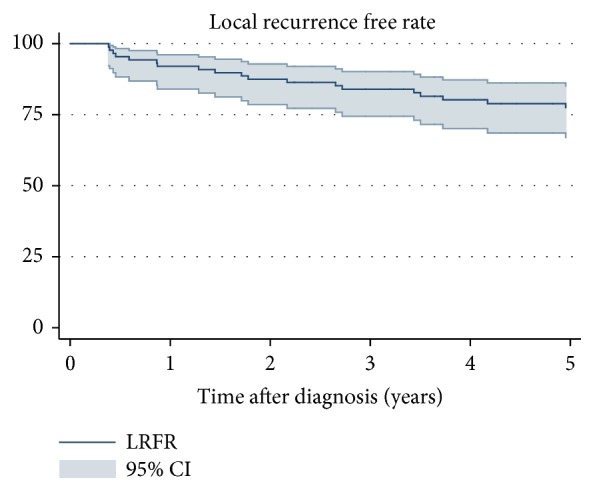
Local recurrence free rate of 88 patients with soft tissue sarcoma in the thoracic wall. 95% confidence intervals.

**Figure 6:**
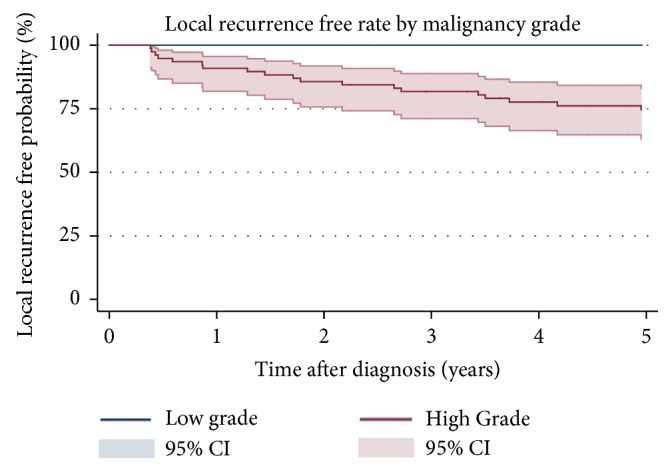
Kaplan-Meier estimates of local recurrence free rate by malignancy grade. 95% confidence intervals.

**Figure 7:**
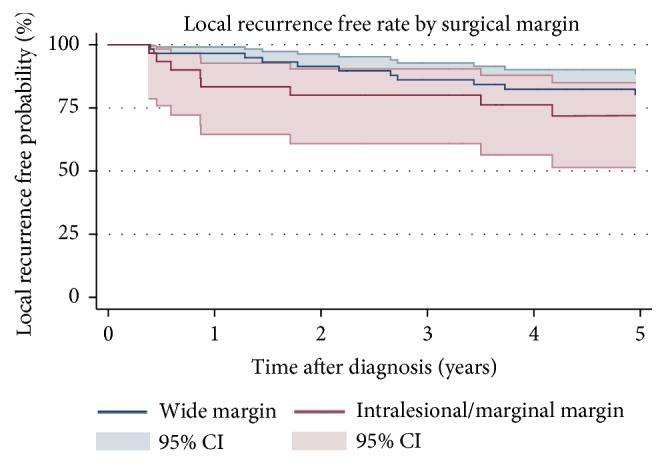
Kaplan-Meier estimates of local recurrence free rate by surgical margin. 95% confidence intervals.

The 5-year LRFR was 77% (95% CI: 0.67-0.85) (Figure 5). Patients with a high grade tumor (5-year LRFR: 75%, 95% CI: 0.63-0.83) had a significant lower 5-year LRFR rate than patients with low grade tumors (Figure 6). LR was more frequent in patients treated with intralesional/marginal (5-year LRFR: 72%, 95% CI: 0.51-0.85) resection compared to wide resection (5-year LRFR: 81%, 95% CI: 0.67.0.89) (Figure 7). Applying univariable and multivariable analysis high grade was associated with worse LRFR, whereas no statistically significant difference between wide and intralesional/marginal resection could be detected (Table 3).

## 4. Discussion

The results of this study establish that OS and LRFR in patients with STS in the thoracic wall are mainly influenced negatively by the malignancy grade, hence tumor biology, and secondly by surgical margin.

### 4.1. Survival

The 5-year OS, 55% (95% CI: 0.44-0.65), is in accordance with a study, including 25 patients treated surgically for a localized STS of the thoracic wall, by Pfannschmidt et al., demonstrating a 5-year OS of 56% [28]. In comparison Gross et al. reported a 5-year survival rate of 87% in chest wall STS, including 55 patients [14]. However, their study population comprised of 41% high grade tumors as opposed to 87% high grade tumors in our study, which could explain the lower mortality. High tumor grade was the main negative predictor of mortality in their multivariate analysis, which has been shown previously and substantiates our results [2, 3, 9, 12, 14, 16–18, 29, 30].

In the present study, the OS rate was remarkably high compared to the number of patients who develop metastases. However, as Figure 1 shows, 37 patients were excluded from the study due to metastases at time of diagnosis. This could be an indicator that chest wall STS tends to metastasize quickly, hence the poor survival rate and furthermore an argument to use chemotherapy for this localization, especially among high grade chest wall sarcomas. A recently published retrospective study by Shewale et al. 121 patients, with sarcomas of the chest wall, found a tendency towards improved mortality when additional systemic therapy was given to high grade tumors at this localization; however it was not statistically significant in their multivariable analysis, only in the univariable analysis [10].

The poor survival rates may be explained by a different age distribution, with more than half of the study population above 50 years and thereby more frequent comorbidity. It has been shown that the level of comorbidity significantly affects both OS and disease-specific mortality in STS patients with localized disease [31]. An adjustment for age was performed in the multivariate analysis, but not comorbidity which therefore might affect our results negatively.

Wide margins are well accepted as important in sarcoma surgery for OS [10], but prior analyses on patients with STS of the thoracic wall have not identified this correlation [12–14, 28]. Common for all of these studies, are the lesser patient cohorts ranging from 25 to 55 patients, which may explain the statistically insignificance. A large study by Salas et al. including 343 patients predominantly with STS on the thoracic wall (83%) (the rest including the abdominal and pelvic wall) found a positive correlation between macroscopically complete surgical resection and OS [29]. Shewale et al. also identified this correlation as statistically significant in their patient cohort consisting of 121 patients with chest wall sarcomas [10]. This current study is, to our knowledge, the first to identify intralesional/marginal surgical resection as a negative predictor of OS in patients with STS of the thoracic wall.

### 4.2. Local Recurrence

Studies including STS of the chest wall have shown 5-year LRFR ranging from 62 to 89% [12, 13]. Tskukushi et al. reported a higher 5-year LRFR (89%) compared to our result (77%) [13]. This might be due to the lower number of high grade tumors and the inclusion of borderline tumors. Supporting this hypothesis is the reduced 5-year LRFR for high grade tumors (75%) and local recurrence only occurred in patients with high grade tumors, thereby making it a prognostic factor for LR.

Surgical margin was not a statistically significant prognostic factor for LR in this study and there were no statistically significant difference among the two groups with regard to 5-year LRFR. Previous studies on chest wall STS have also not been able to identify this correlation [11, 12, 14]. McMillan et al. proposed that even though it is commonly accepted that incomplete resection will result in a higher degree of local recurrence the finding in these studies may be due to a low sample size or reflect the effect of adjuvant therapies [11]. The reason for this outcome remains a paradox to us.

Consistent with previous studies most LRs occur within the first year after surgery [11, 16, 32]. This may support the results of a recently published study, suggesting more intense surveillance of high malignant sarcomas within the first two years after surgery to detect more local recurrences and lung metastases [32].

### 4.3. Comparison with Extremity STS

Studies of extremities STS have shown 5-year survival rates ranging from 67-76% [3, 17, 30]. A previous study by Vraa et al. included 152 patients, with STS of the thigh in the period 1979-1998, [17]. They found a 5-year survival rate of 67% and a 5-year local control rate of 91%. Malignancy grade was a prognostic factor for survival and LR, whereas surgical margin only influenced LR. Their study population comprised of 82% intermediate/high grade tumors, compared to 87% in this current study. In more than half of the cases in this study (66%) wide margin was achieved, as opposed to 51% in the study by Vraa et al. In addition, we also had a larger percentage of patients treated with radiotherapy (34% versus 21%) and chemotherapy (15% versus 3%). These differences among the patient cohort could affect the OS and LRFR, with malignancy grade as the main predictor. Adjusting for radiotherapy and chemotherapy in the multivariate analysis had no statistically significant impact on OS or LRFR.

Few studies indicate that sarcomas of the chest wall and of the extremities have a similar prognosis [1, 13], while other studies report a lower OS rate for thoracic wall sarcomas [9, 15]. The variety in survival rates between thoracic wall sarcomas and extremities sarcomas could be explained by the anatomic characteristics, with no clear anatomic boundaries and compartments making it increasingly difficult to resect a large tumor with an adequate margin in the thoracic wall compared to a tumor in the extremities [12, 13]. Gutierrez et al. showed that tumor site was a prognostic factor for survival, with higher mortality for STS of the thoracic wall compared to STS of the extremities [9]. Another explanation, aside from treatment difficulty, might be the difference in tumor biology affected by localization. Dasgupta et al. reported how the mortality for rhabdomyosarcomas is influenced by localization, with poor prognosis for paranasal site and extremities compared to orbital rhabdomyosarcoma with good prognosis [22]. This might indicate that STS of the chest wall exhibit different biology compared to STS of the extremities.

As opposed to studies including STS of the chest wall, studies including STS of the extremities have found wide surgical margin as a predictor of better local control [2, 3, 17, 20, 30, 33, 34]. In another study by Stojadinovic A. et al. tumor site was found to be a prognostic factor for LR. This supports our result and the previous comparison with the study by Vraa et al. Local spread may also be related to type of tissue and type of sarcoma; however the exact reason for the difference remains unclear, which calls for further investigations.

### 4.4. Methodological Considerations

This present study is to our knowledge one of the largest studies to date focusing exclusively on STS of the thoracic wall [12, 14, 28]. We did not include borderline tumors and certain other histological tumors to investigate a more homogenous group and to avoid an overestimation of the survival rate, even though a few previous studies did, thereby making a directly comparison difficult [11, 13, 16]. The limitations of this study include the retrospective design, making it more susceptible to bias and confounding, and due to the predefined collection of information, the data may be less specific regarding the question in focus compared to a prospective study. On the other hand, using reliable population based databases (ASR and DSR) has several advantages including the large number of patients and ensuring that data is collected prospectively without relation to a specific study preventing the risk of differentiated misclassification. Furthermore, ASR has been validated regarding the data registered as well as the completeness of registration [35]. Another limitation of this study is the comparison with the study by Vraa et. al., seeing a strict comparison between the two studies is not possible. However, both studies are using the same database, ASR, and are carried out at the same institution, where the standard treatment regime for chest wall and extremity STS is the same [35]. There has been a shift in treatment protocol during the years, with a tendency towards “closer” surgical removal and adjuvant radiotherapy, making direct comparisons to the study by Vraa et al. difficult as their study is from 1979 to 1998 [17, 35]. Maretty et al. performed a large database study, using ASR, focusing on STS of the chest wall and extremities; they found no significant change in disease-specific mortality and LR due to this shift in treatment regime [35].

To ensure high extern validity three pathologists performed the histopathologically analysis, based on Myhre Jensen and FNCLCC [26, 27]. A previous study found limited discrepancies between the two scales; therefore, we expect the comparability issues to be minor [2].

Intralesional and marginal resection were joined into a single group, since preliminary analysis did not reveal any difference between the two groups in terms of mortality and local control.

## 5. Conclusion

High malignancy grade was identified as a negative prognostic factor for OS and LR. A positive surgical margin influenced OS negatively, but not LRFR. STS of the thoracic wall showed lower OS and LRFR compared to STS of the extremities, indicating a change in treatment protocol among the two groups is needed. Further research is needed to investigate the differences in tumor biology depending on localizations.

## Figures and Tables

**Table 1:** Patient, tumor, and treatment characteristics.

Factors	n (%)
Age, years	
16-49	29 (33%)
≥ 50	59 (67%)
Sex	
Male	51 (58%)
Female	37 (42%)
Tumor size^a^	
1-4 cm	37 (44%)
5-9 cm	28 (28%)
≥ 10 cm	20 (28%)
Tumor depth	
Superficial	32 (36%)
Deep	56 (64%)
Malignancy grade	
Low	11 (13%)
Intermediate/high	77 (87%)
Surgical margin	
Wide	58 (66%)
Intralesional/marginal	30 (34%)
Chemotherapy	
Yes	13 (15%)
No	75 (85%)
Radiotherapy	
Yes	30 (34%)
No	58 (66%)
Local recurrence^b^	
Yes	18 (20%)
No	69 (80 %)
Distant metastases^b^	
Yes	18 (20%)
No	69 (80%)

^a^Data missing for 4 patients.

^b^Data missing for 1 patient.

**Table 2:** Histological types of soft tissue sarcomas.

Histological type	Number	%
UPS*∗*	21	24
Leiomyosarcoma	19	22
Liposarcoma	11	12
Fibrosarcoma	5	6
Malignant schwannoma	6	7
Synovial sarcoma	7	8
Unclassifiable	8	9
Other types	11	12

Total	88	100

*∗*Undifferentiated pleomorphic sarcoma.

**Table 3:** Analyses of unfavorable prognostic factors for survival and local control.

	Overall survival		Local recurrence free rate	
Factors	HR		HR	
	Crude	Adjusted^a^	Crude	Adjusted^a^
	(95% CI)	(95% CI)	(95% CI)	(95% CI)
Resection				
Marginal/intralesional	4,54 (2.44-8.45)	3,26 (1.30-8.16)	1,97 (0.97-4.00)	1,24 (0,43-3,60)
Malignancy grade				
High	-^b^	-	-^c^	-

HR: hazard ratio.

CI: confidence interval.

^a^Resection was adjusted for age, sex, tumor size, depth, malignancy grade, radiotherapy, and chemotherapy. Malignancy grade was adjusted for age, sex, tumor size, depth, resection type, radiotherapy, and chemotherapy.

^b^None of the patients with low grade tumors died.

^c^Local recurrence only occurred in patients with high grade tumors.

## Data Availability

Data from the Aarhus Sarcoma Registry and Danish Sarcoma Registry are only available for researchers and institutions who meet the criteria for access to confidential data. Future researchers will be able to access the data through the same process which the authors of this manuscript did: authorization to manage and process data: Danish Data Protection Agency, https://www.datatilsynet.dk/english/the-danish-data-protection-agency/introduction-to-the-danish-data-protection-agency/, e-mail: dt@datatilsynet.dk; application for data: the Danish Sarcoma Registry: http://www.rkkp.dk/in-english/, http://www.rkkp.dk/forskning/, e-mail: fagligkvalitet@rkkp.dk.
